# Rhein Inhibits Cell Development and Aflatoxin Biosynthesis via Energy Supply Disruption and ROS Accumulation in *Aspergillus flavus*

**DOI:** 10.3390/toxins16070285

**Published:** 2024-06-23

**Authors:** Xiaoyan Wang, Kashif Iqbal Sahibzada, Ruibo Du, Yang Lei, Shan Wei, Na Li, Yuansen Hu, Yangyong Lv

**Affiliations:** 1 College of Biological Engineering, Henan University of Technology, Zhengzhou 450001, China; wangxiaoyan990416@163.com (X.W.); kashif.iqbal@dhpt.uol.edu.pk (K.I.S.); drb17719229855@163.com (R.D.); leiyang@haut.edu.cn (Y.L.); weishansd2014@163.com (S.W.); nalinwsuaf@haut.edu.cn (N.L.); hys308@126.com (Y.H.); 2 Department of Health Professional Technologies, Faculty of Allied Health Sciences, The University of Lahore, Lahore 54570, Pakistan

**Keywords:** rhein, *Aspergillus flavus*, AFB_1_ biosynthesis, transcriptomics

## Abstract

*Aspergillus flavus* and its carcinogenic secondary metabolites, aflatoxins, not only cause serious losses in the agricultural economy, but also endanger human health. Rhein, a compound extracted from the Chinese herbal medicine *Rheum palmatum* L. (Dahuang), exhibits good anti-inflammatory, anti-tumor, and anti-oxidative effects. However, its effect and underlying mechanisms against *Aspergillus flavus* have not yet been fully illustrated. In this study, we characterized the inhibition effect of rhein on *A. flavus* mycelial growth, sporulation, and aflatoxin B_1_ (AFB_1_) biosynthesis and the potential mechanism using RNA-seq analysis. The results indicate that *A. flavus* mycelial growth and AFB_1_ biosynthesis were significantly inhibited by 50 μM rhein, with a 43.83% reduction in colony diameter and 87.2% reduction in AFB_1_ production. The RNA-seq findings demonstrated that the differentially expressed genes primarily participated in processes such as spore formation and development, the maintenance of cell wall and membrane integrity, management of oxidative stress, the regulation of the citric acid cycle, and the biosynthesis of aflatoxin. Biochemical verification experiments further confirmed that 50 μM rhein effectively disrupted cell wall and membrane integrity and caused mitochondrial dysfunction through disrupting energy metabolism pathways, leading to decreased ATP synthesis and ROS accumulation, resulting in impaired aflatoxin biosynthesis. In addition, a pathogenicity test showed that 50 μM rhein inhibited *A. flavus* spore growth in peanut and maize seeds by 34.1% and 90.4%, while AFB_1_ biosynthesis was inhibited by 60.52% and 99.43%, respectively. In conclusion, this research expands the knowledge regarding the antifungal activity of rhein and provides a new strategy to mitigate *A. flavus* contamination.

## 1. Introduction

*Aspergillus flavus* has been found to contaminate peanuts, maize, and wheat during cultivation, storage, processing, and transportation [1,2]. *A. flavus* also produces highly carcinogenic secondary metabolites called aflatoxins, of which aflatoxin B_1_ is classified as a grade 1 carcinogen [3,4]. The consumption of aflatoxin-contaminated food can cause poisoning, which may lead to hepatocellular carcinoma, seriously endangering the life and health of both humans and livestock [5]. Studies have shown that over 120 different countries have established maximum permissible levels of aflatoxin contamination in food products [6]. It is therefore important to develop rapid, efficient, and safe measures to address the problems associated with AFB_1_ contamination, given the serious threat it poses to human health.

Biological, physical, and chemical methods have been employed to prevent the contamination of *A. flavus* in food [7]. Biological measures, such as endophytic *Aspergillus fumigatus* and lactic acid bacteria, are efficient in inhibiting the growth of *A. flavus* and lessening mycotoxin production [8,9]. Physical methods, such as γ-irradiation of maize artificially inoculated with *A. flavus*, also resulted in a reduction in aflatoxins. In recent years, compounds from natural plants, including cinnamaldehyde, citral, and eugenol, have shown excellent antifungal performance against *A. flavus* and aflatoxin biosynthesis, owing to their eco-friendly properties [10]. Therefore, it is imperative to develop natural compounds to effectively prevent *A. flavus* growth and aflatoxin contamination.

Rhein, an anthraquinone compound derived from the natural plant Rhubard, also known as a kind of traditional Chinese herbal medicine, exhibits various effects, including anti-inflammatory, anti-tumor, antioxidant, anti-diabetic, cardioprotective, neuroprotective, hepatoprotective, and nephroprotective properties [11,12,13]. For example, rhein inhibits colon cancer cell growth by targeting the mammalian target of rapamycin (mTOR) pathway and human kidney cancer cells through the mitogen-activated protein kinase (MAPK)/nuclear factor kappa-light-chain enhancer of activated B cells (NF-κB) signaling pathway [14,15]. Additionally, studies have demonstrated that rhein can inhibit *Propionibacterium acnes* growth through inhibiting NADH dehydrogenase-2 activity [16]. Previous reports have also shown that rhein and its derivatives exhibit insecticidal and antifungal activity against *Saprolegnia* sp., *Phytophthora capsici*, *Candida albicans*, *Cryptococcus neoformans*, and *Trichophyton mentagrophytes* [17,18,19,20,21,22,23]. However, the effects and underlying mechanisms of rhein against *A. flavus* mycelium growth and aflatoxin biosynthesis have not yet been investigated.

In the current study, we investigated the effects of rhein on *A. flavus* mycelium growth, spore formation, and AFB_1_ biosynthesis. Rhein was found to significantly diminish the pathogenicity of *A. flavus* on both maize and peanut seeds. An analysis of the transcriptomic data showed that differential gene expression was involved in conidium formation and development (*wetA*, *BrlA*, *AbaA*), cell wall and membrane structure, oxidative stress response, the citrate cycle, and aflatoxin biosynthesis (*aflA*, *aflC*, *aflO*, *aflQ*). Our study provides new strategies for the prevention of aflatoxin contamination.

## 2. Results

### 2.1. Effect of Rhein on A. flavus Growth, Spore Formation, and AFB_1_ Production

**Figure 1:**
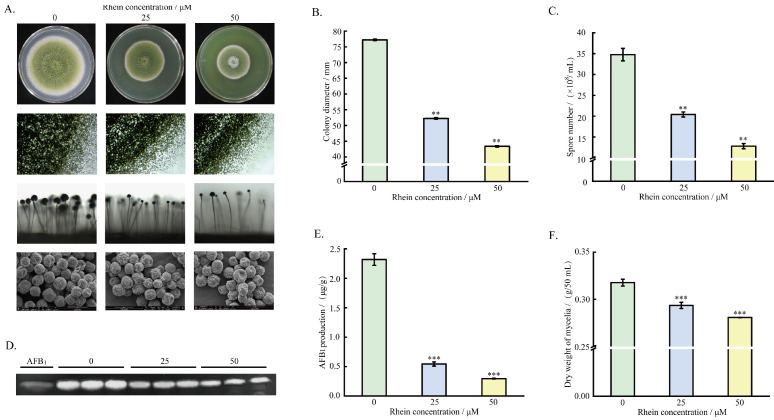
Effects of rhein (0, 25, 50 μM) on *A. flavus* morphology and AFB_1_ biosynthesis. (**A**) Observation of colony and conidial morphology; (**B**) colony diameter; (**C**) the number of spores; (**D**) AFB_1_ production according to TLC analysis; (**E**) AFB_1_ production according to HPLC analysis; and (**F**) dry weight of mycelia. ** represents *p* < 0.01, *** represents *p* < 0.001.

To examine the antifungal effects of rhein on mycelium growth and AFB_1_ production, 2 μL of *A. flavus* spore solution (1 × 10^6^ spores/mL) was inoculated into PDA medium with varying concentrations of rhein (0, 25, or 50 μM), then incubated at 30 °C for 5 days. The results demonstrate that *A. flavus* mycelium growth was significantly inhibited by treatment with 25 and 50 µM rhein. Additionally, the mycelium became dense and the colony diameter was reduced by 32.34% and 43.83%, respectively. Regarding the formation of *A. flavus* spores, it was observed that the number of spores and conidiophores decreased significantly with increasing rhein concentration, where the number of spores was significantly reduced by 41.32% and 63.18% under the 25 and 50 μM rhein treatments, respectively. Electron microscopy analysis revealed that the surface of the conidia without rhein treatment was regular and the shape was plentiful, while the morphology of rhein-treated *A. flavus* spores appeared shrunken and irregular (Figure 1A–C). TLC and HPLC analysis showed that, compared to the control, AFB_1_ production decreased by 76.5% and 87.2% after treatment with 25 and 50 μM rhein, respectively, suggesting a dose-dependent inhibition of AFB_1_ biosynthesis (Figure 1D,E). Additionally, the dry weight of mycelia also decreased significantly with increasing rhein concentration (Figure 1F). In summary, rhein showed significant inhibitory effects against *A. flavus* mycelium growth, spore formation, and AFB_1_ yield; moreover, 50 μM rhein showed better inhibition of *A. flavus* mycelium growth, sporulation, and AFB_1_. Based on these results, the *A. flavus* treated with 50 μM rhein was selected for RNA-seq analysis.

### 2.2. Transcriptomic Analysis

**Figure 2:**
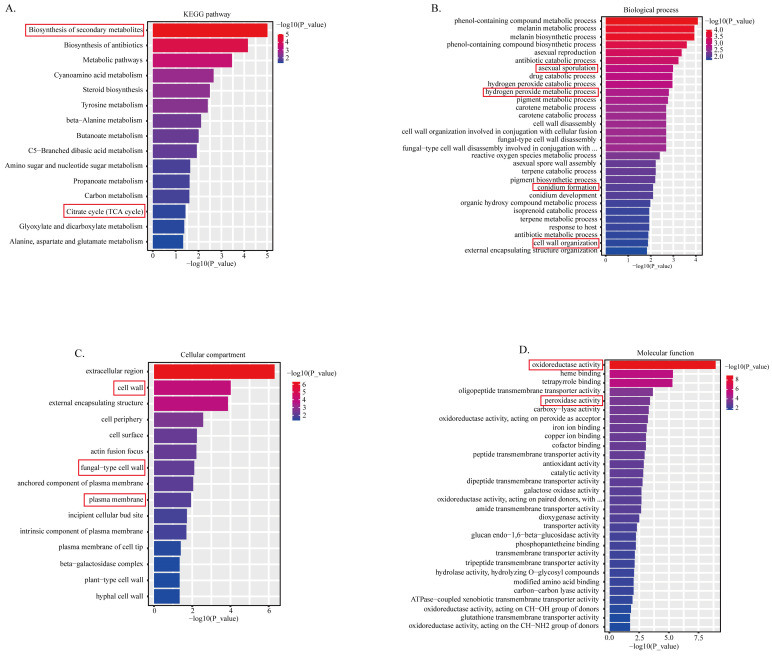
Transcriptome analysis of rhein-treated and untreated *A. flavus* mycelia. (**A**) KEGG pathway enrichment; (**B**) enrichment of biological processes; (**C**) analysis of the enrichment of cellular components in DEGs; and (**D**) analysis of DEGs for molecular function enrichment. The red boxes indicated the related items in Table 1 and the subsequent biochemical verification experiments.

The *A. flavus* treated with 50 μM rhein was selected for RNA-seq analysis with triple biological replicates in order to explicate the underlying mechanism through which rhein affects *A. flavus* mycelium growth and aflatoxin biosynthesis. The results show 633 differentially expressed genes (DEGs) in rhein-treated *A. flavus*, with 150 DEGs upregulated and 483 DEGs downregulated compared to the control. To further elucidate the function of the DEGs, enrichment analysis was conducted. The results reveal that the DEGs were primarily linked to the biosynthetic pathway of secondary metabolites, metabolic pathways, and the citric acid cycle, as indicated by the KEGG enrichment analysis (Figure 2A). GO enrichment analysis revealed that DEGs were associated with conidium formation and development, the cell wall and membrane, the hydrogen peroxide metabolic process, and oxidoreductase activity (Figure 2B–D).

To better elucidate the underlying mechanism, the representative DEGs were classified into four categories, including conidium formation and development, cell wall and cell membrane, glycolysis, citrate cycle, oxidative stress, and aflatoxin biosynthesis (Table 1).

### 2.3. Effect of Rhein on the Hydrophobicity of A. flavus

**Figure 3:**
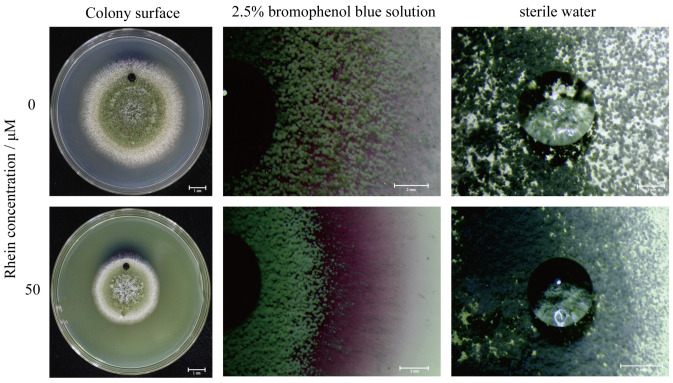
Determination of the hydrophobicity of *A. flavus* treated with or without rhein.

To verify the differential expression of conidial hydrophobin genes, the effect of rhein on the hydrophobicity of *A. flavus* was investigated. The results demonstrate that bromophenol blue solution and sterile water fully penetrated the surface of the rhein-treated *A. flavus* mycelium into the medium as compared to the control. The mycelial surface was observed using a stereoscopic microscope, and at the same magnification, the sterile water droplets on the surface of *A. flavus* colonies treated with rhein were found to be smaller (Figure 3). These results show that treatment with rhein inhibits the hydrophobicity of the cell surface of the mycelia.

### 2.4. Effect of the Rhein on the Integrity of A. flavus Cell Wall

**Figure 4:**
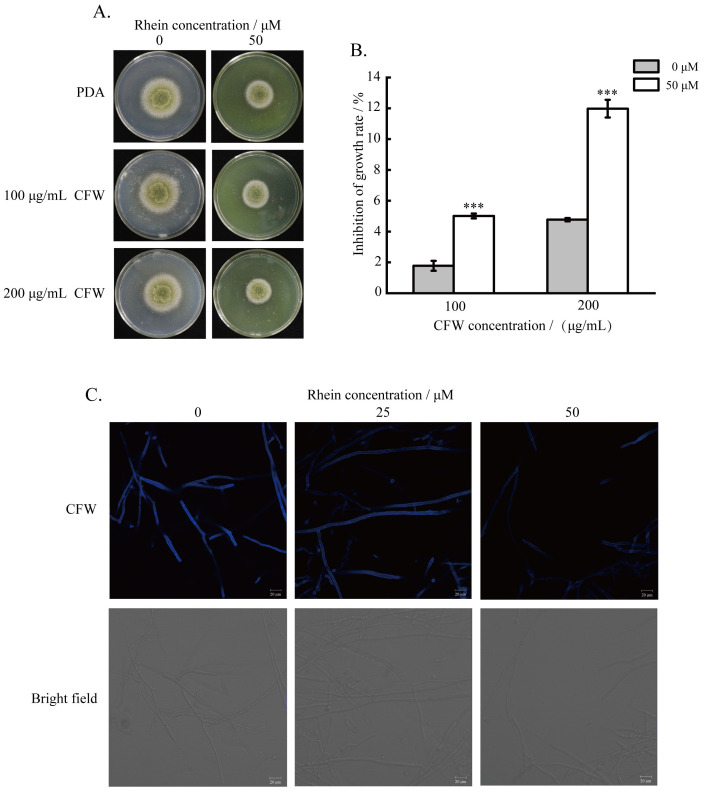
Effects of rhein on *A. flavus* cell wall. (**A**) The sensitivity of *A. flavus* to CFW after treatment with rhein; (**B**) the CFW inhibition of *A. flavus* mycelial growth; (**C**) *A. flavus* mycelia treated with rhein (0, 25, 50 μM) stained with CFW by CLSM. *** represents *p* < 0.001.

To study the effect of rhein on *A. flavus* cell wall integrity, the strains were cultured for 4 days under the reagent CFW. The results unveil that *A. flavus* strains were more sensitive to CFW after rhein treatment than the controls (Figure 4A,B). Additionally, the integrity of the cell wall was examined through CFW staining, showing a decrease in CFW fluorescence intensity with increasing rhein concentration (Figure 4C). These results suggest that rhein disrupts *A. flavus* cell wall synthesis.

### 2.5. Effect of Rhein on the Integrity of Cell Membrane

**Figure 5:**
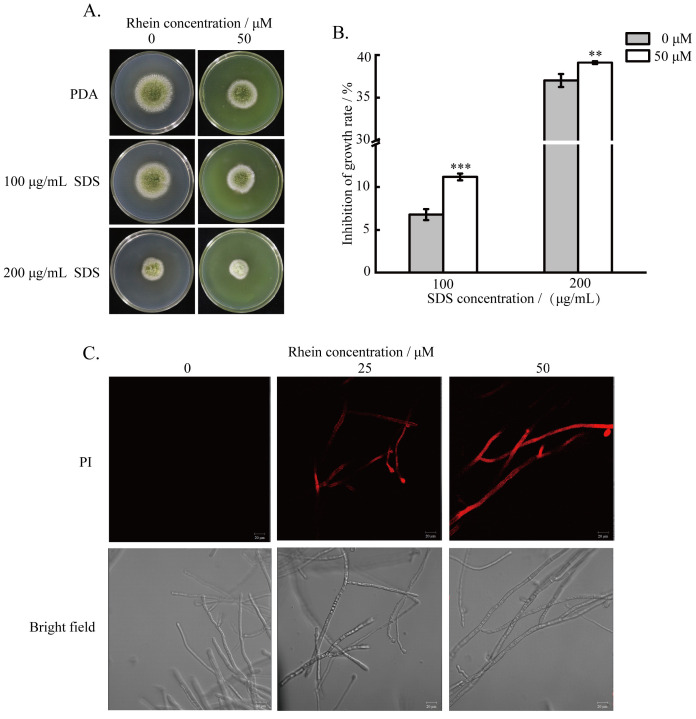
Effects of rhein on A. flavus cell membrane. (**A**) The sensitivity to SDS of A. flavus treated with rhein; (**B**) the SDS inhibition of A. flavus mycelial growth; (**C**) A. flavus mycelial treated with rhein (0, 25, 50 μM) stained with PI by CLSM. ** represents *p* < 0.01, *** represents *p* < 0.001.2.6. Effects of Rhein on Intracellular ROS, H_2_O_2_, and ATP in A. flavus.

Different concentrations of SDS were introduced into PDA medium to examine the effect of rhein on the integrity of *A. flavus* cell membranes. The results demonstrate that the inhibition rate of rhein-treated *A. flavus* reached 11.2% and 39.1% under the conditions of 100 and 200 μg/mL SDS, respectively. Rhein-treated *A. flavus* was more sensitive than the control group (Figure 5A,B). Additionally, PI staining revealed that the red fluorescence intensity gradually increased with the concentration of rhein (Figure 5C). These results reveal that the addition of rhein disrupts the cell membrane of *A. flavus*.

**Figure 6:**
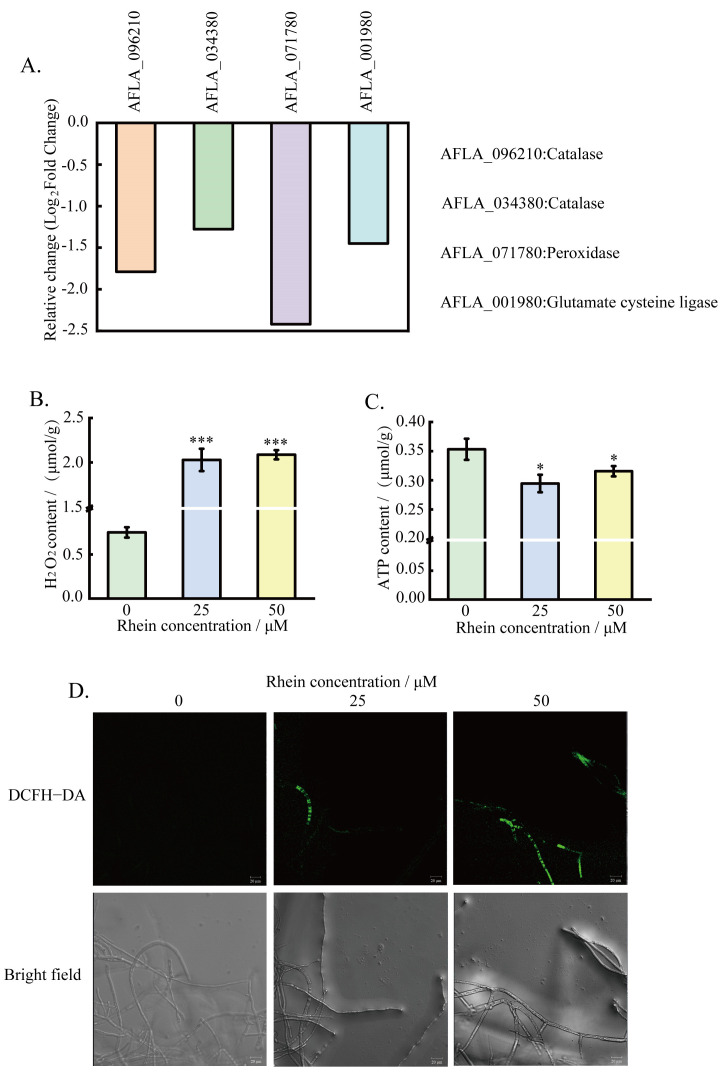
Effects of rhein on *A. flavus* intracellular ROS. (**A**) Expression levels of differential genes associated with oxidative stress in *A. flavus* after rhein treatment; (**B**) H_2_O_2_ content; (**C**) ATP content; (**D**) *A. flavus* mycelial treated with rhein (0, 25, 50 μM) stained with DCFH-DA by CLSM. * represents *p* < 0.05, *** represents *p* < 0.001.

To validate the analysis of RNA-seq data, the intracellular ROS accumulation, H_2_O_2_, and ATP contents were measured. The results demonstrate that, accompanied by the downregulated expression of catalase, peroxidase, and glutamate cysteine ligase-related genes (Figure 6A), the intracellular H_2_O_2_ content of *A. flavus* increased (Figure 6B), which was in agreement with the results of the transcriptome analysis. The intracellular ATP content of *A. flavus* also decreased slightly after rhein treatment (Figure 6C). In addition, the results of DCFH-DA staining demonstrate that *A. flavus* mycelia treated with rhein exhibited stronger green fluorescence than those untreated with rhein (Figure 6D), indicating that rhein induced the accumulation of ROS.

### 2.6. Effect of the Rhein on A. flavus Pathogenicity on Crop Seeds

**Figure 7:**
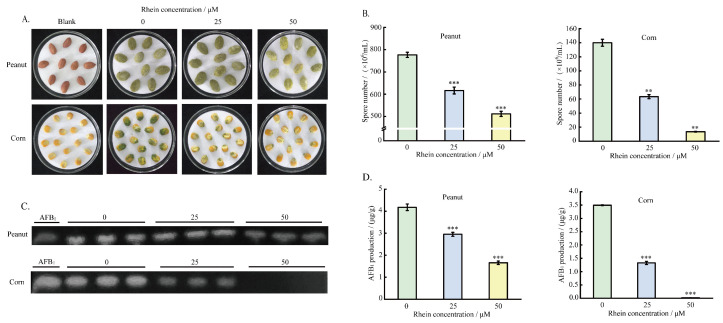
Effect of rhein on *A. flavus* pathogenicity to crop seeds. (**A**) Growth of *A. flavus* on seeds with or without rhein treatment; (**B**) number of *A. flavus* spores on seeds; (**C**) TLC; and (**D**) HPLC analysis of AFB_1_ yield. ** represents *p* < 0.01, *** represents *p* < 0.001.

To investigate the effect of rhein on the sporogenesis and the AFB_1_ yield of *A. flavus* on crop seeds, different concentrations of rhein were incubated for 5 days with maize and peanut seeds infected with *A. flavus* spore suspension. The results indicate that the colonization ability of *A. flavus* on peanut and maize seeds was reduced after treatment with rhein (Figure 7A). The number of spores on the surface of the seeds was reduced by 90.4% and 34.1%, respectively, in comparison to the control group, following treatment with 50 μM of rhein (Figure 7B). Additionally, the TLC and HPLC results demonstrate the inhibition of AFB_1_ production. With the 50 μM rhein treatment, the AFB_1_ yield of *A. flavus* on peanuts and maize decreased by 60.52% and 99.43%, respectively (Figure 7C,D). These results clearly demonstrate that rhein significantly inhibits the pathogenicity of *A. flavus* on maize and peanut seeds.

## 3. Discussion

Developing safe and effective antifungal drugs is urgently needed to reduce the threat posed by *A. flavus* to cereals and food. In our study, the effects of rhein, a compound from traditional Chinese medicine, on *A. flavus* mycelium growth, sporulation, and AFB_1_ biosynthesis were characterized. The results demonstrate that rhein disrupted *A. flavus* cell wall and membrane integrity, thereby inhibiting its growth and sporulation. Additionally, an RNA-seq analysis and subsequent validation demonstrated that mitochondrial dysfunction and the accumulation of intracellular ROS might lead to the inhibition of aflatoxin biosynthesis.

Rhein treatment significantly inhibited cell growth and development in *A. flavus*. Various regulators and transcription factors regulate the *A. flavus* spore-forming process. It has been reported that conidia formation and maturation in *A. flavus* are mainly governed by BrlA, AbaA, and WetA [24]. The *AbaA* gene plays a role in conidial cell differentiation and is activated by BrlA at metaphase [25,26], while the *wetA* gene, activated by *AbaA* in anaphase, participates in synthesizing key conidial cell wall components [27]. In our study, rhein treatment substantially reduced the *A. flavus* spore count, and the expression of genes related to conidial growth, such as *AFLA_052030* (*wetA*), *AFLA_082850* (*BrlA*), *AFLA_029620* (*AbaA*), and *AFLA_044790*, was significantly downregulated (Table 1). These findings suggest that rhein may inhibit spore formation in *A. flavus*. In addition, RodA and RodB are conidial surface proteins, with RodA playing a role in the surface rod structure and RodB contributing to the conidial cell wall structure in *Aspergillus fumigatus* [28,29]. In our study, the bromophenol blue solution diffused rapidly and was absorbed on the surface of rhein-treated mycelium (Figure 3). Furthermore, the expression of genes related to hydrophobic proteins (*AFLA_098380*, *AFLA_014260*) was significantly downregulated. This is in agreement with the results of the hydrophobicity assay in the *Afper1* gene deletion strain studied by Lv et al. [30]. These results suggest that rhein treatment may have reduced the hydrophobicity of the mycelium surface.

The cell wall and membrane of the fungi are dynamic structures which are essential for maintaining cell viability and integrity, making them promising targets for antifungal treatment [31,32,33,34,35]. In this study, CFW staining showed that rhein disrupted cell wall synthesis, and genes involved in cell wall protein synthesis (*AFLA_061960*) were also downregulated. However, transcriptome data analysis demonstrated that gene expression of chitin synthetase was upregulated, suggesting that upregulated chitin synthetase might contribute to compensating for the destroyed cell wall after rhein treatment. In addition, PI staining demonstrated that the red fluorescence intensity was increased, suggesting cell membrane damage. Similar phenomena have been observed in *A. flavus* cell membranes when treated with honokiol [36], (E)-2-hexenal [37], and phenyllactic acid [38]. Moreover, the gene expression levels of *AFLA_007630* and *AFLA_039810*, which are involved in membrane protein synthesis, were significantly downregulated. This suggests that rhein may induce impaired synthesis of the cell wall and membrane, leading to the disruption of cell components. These results also indicate that the antifungal activity of rhein may target the integrity of the cell membrane and cell wall.

It is well known that mitochondria are organelles that generate ATP through oxidative phosphorylation reactions, providing energy to the cell [39]. Oxidative phosphorylation occurs during the citric acid cycle (TCA) in the mitochondria [40]. Previous studies have indicated that mitochondrial dysfunction leads to the accumulation of ROS by reducing ATP levels [41]. In our study, genes associated with the TCA cycle, including citrate synthetase and succinyl-CoA synthetase, were significantly downregulated after rhein treatment (Table 1). This finding mirrors the genetic changes observed in *A. flavus* treated with estragole [42]. In addition, *A. flavus* treated with rhein exhibited a remarkable decrease in intracellular ATP levels, which aligns with the findings of Duan et al. [43] regarding *A. flavus* treated with 1-octanol. These results reveal that rhein affects the changes in enzymes in other metabolic pathways, such as the TCA cycle, leading to mitochondrial dysfunction and ultimately the accumulation of ROS.

Peroxisomes are an important component of eukaryotic cells, participating in various cellular physiological processes and being capable of rapidly producing and removing H_2_O_2_ and O_2_, thereby regulating the dynamic changes in ROS levels [44,45,46]. In this study, the genes encoding catalase and peroxidase (*AFLA_096210*, *AFLA_034380*) were significantly downregulated after treatment with rhein (Figure 6A). Subsequently, experimental verification showed that the intracellular H_2_O_2_ content was increased in *A. flavus* treated with rhein. These results suggest that H_2_O_2_ accumulation disrupts oxidation reduction (redox) homeostasis. In addition, glutathione biosynthesis is also involved in oxidative stress, with glutathione ligase reported as the key enzyme catalyzing glutathione biosynthesis [47]. Elevated levels of ROS also promote mitochondrial and metabolic dysfunction, resulting in oxidative stress [48]. Our transcriptome data show that the transcriptional level of the glutathione ligase gene (*AFLA_001980*) was remarkably downregulated (Table 1). We also assessed the intracellular ROS level in *A. flavus* using DCFH-DA staining and noted the dose-dependent increase in fluorescence intensity with rhein treatment. These results suggest that rhein triggered oxidative stress, resulting in a significant accumulation of ROS, consistent with the intracellular ROS level observed in *A. flavus* treated with methyl 2-methylbutyrate [49]. Therefore, we speculate that rhein treatment might cause changes in the intracellular peroxisomes of *A. flavus*, which disrupted the redox balance and subsequently led to ROS accumulation.

Aflatoxin biosynthesis involves a complex regulatory network composed of multiple genes and enzymatic reactions, regulated by the pathway-specific transcription factor aflR and several structural genes, including *aflA*, *aflB*, *aflC*, *aflO*, *aflQ*, *aflR*, and *aflS*. Additionally, it is also governed by environmental conditions, such as light, nutrient sources, and oxidative stress [50,51,52,53]. According to previous reports, three proteins, aflA, aflB, and aflC, are jointly involved in the synthesis of norsolorinic acid (NOR) in the biosynthesis pathway of aflatoxin [5]. AflO, which encodes O-methyltransferase, converts demethylsterigmatocystin (DMST) to a new compound, sterigmatocystin (ST), and aflQ is a P-450 monooxygenase that converts O-methylsterigmatocystin (OMST) to AFB_1_ [3]. In our study, the production of AFB_1_ by rhein-treated *A. flavus* growing on both PDA medium and grain was significantly decreased. Analysis of transcriptome data revealed that the genes involved in the pathway for the aflatoxin synthesis (*aflA*, *aflC*, *aflO,* and *aflQ*) were all remarkedly downregulated after rhein treatment. In *A. flavus*, deletion of catalase CTA1 contributes to the intracellular ROS accumulation and downregulation of *aflC*, *aflD*, and *aflQ,* consequently leading to impaired AFB_1_ biosynthesis [54]. Additionally, the accumulated intracellular ROS in *A. flavus* treated with curcumin also leads to a reduction in aflatoxin production [55]. In our study, the downregulated expression levels of *aflA*, *aflC*, *aflO,* and *aflQ* and decreased AFB_1_ biosynthesis may be attributed to the ROS accumulation triggered by rhein treatment.

## 4. Conclusions

**Figure 8:**
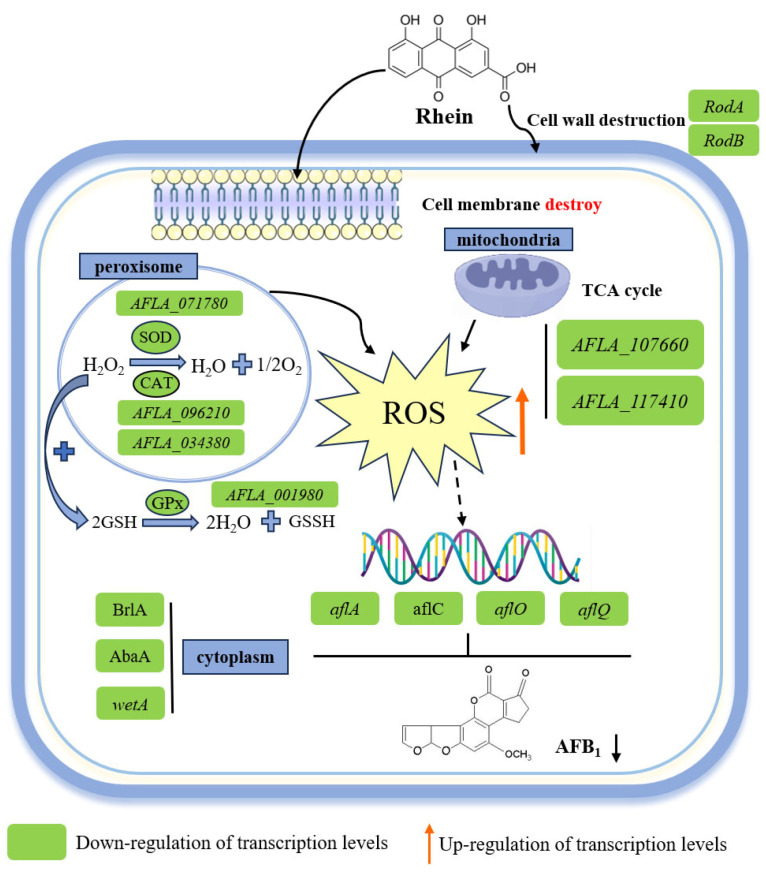
Mechanistic diagram of rhein inhibiting *A. flavus* growth and AFB_1_ biosynthesis.

In summary, rhein inhibited the growth and development of *A. flavus* by interfering with cell wall and membrane synthesis. Additionally, rhein might disrupt energy metabolism, cause mitochondrial dysfunction, and decrease ATP synthesis, leading to ROS accumulation, thereby inhibiting AFB_1_ biosynthesis (Figure 8). Furthermore, rhein treatment effectively inhibited mycelial growth and the production of AFB_1_ in both peanut and maize seeds. Our findings indicate that rhein can be considered as a potential novel agent for preventing fungal infections in food and feedstuffs.

## 5. Materials and Methods

### 5.1. Strains, Chemicals, and Medium

The model strain *A. flavus* NRRL 3357, a kind gift from Prof. Zhumei He (Sun Yat-sen University, China), was used in this study. Rhein (CAS:478-43-3, 98.5% purity) was obtained from J&K Scientific Ltd. (Beijing, China). Peanut and maize seeds were sourced from local markets. Potato dextrose agar medium (PDA) was selected for fungal growth, conidial production, and aflatoxin B_1_ (AFB_1_) biosynthesis. Rhein was dissolved in DMSO to form a solution with a final concentration of 10 mg/mL.

### 5.2. Effect of Rhein on Phenotype, Spore, and Aflatoxin B_1_ Production of A. flavus

The effects of rhein on *A. flavus* colony size, spore morphology, and aflatoxin B_1_ yield were examined with PDA medium. Rhein at different concentrations (25 and 50 μM) was added to PDA medium to create treatment groups according to previously described methods [56], while the same volume of DMSO was added to the control group. The spore solution (1 × 10^6^ spores/mL) was inoculated, and the colony diameter was recorded after culturing at 30 °C for 5 days. The morphology of spores and conidial pedicles was observed using a light microscope, stereoscopic microscope, and electron microscope.

AFB_1_ was extracted and detected using the methods described in a previous study [42]. In brief, 25 μM or 50 μM of rhein were introduced into PDA medium, and 2 μL of spore solution was inoculated followed by incubation for 5 days at 30 °C. AFB_1_ was then extracted by adding chloroform and distilled water (volume ratio of 1:1). Finally, AFB_1_ was analyzed through HPLC and TLC.

To evaluate the impact of rhein on mycelium production, 25 or 50 μM of rhein was introduced into liquid medium containing 50 mL DPY. Subsequently, *A. flavus* spores were inoculated to achieve a concentration of 1 × 10^6^ spores/mL. After shaking for 3 days at 30 °C, the mycelium was filtered and dried for 6 h, then weighed.

### 5.3. RNA-seq Analysis

The *A. flavus* spore suspension and 50 μM rhein were grown in PDA medium for 60 h at 30 °C, with DMSO replacing rhein in the control group. The TRIzol method by Thermo Fisher, Shanghai, China, was employed to extract total RNA from the mycelia with three biological replicates, and the quality and integrity of RNA were assessed. Subsequently, Shanghai Yuanxin Biotechnology Co., Ltd. (Shanghai, China) constructed the cDNA library for RNA-seq analysis. Each sample with three biological replicates, |log_2_fold change| > 1, and *p* value < 0.05 was defined as a differentially expressed gene. KEGG enrichment analysis and GO were performed using Goatools and KOBAS software, respectively. Raw transcriptome data were entered into the CNCB database under CRA accession number CRA015824.

### 5.4. Effect of Rhein on Hydrophobicity of A. flavus

Hydrophobicity was determined using the method of a previous study with slight modifications [57]. *A. flavus* spores were point-inoculated on PDA medium supplemented with rhein (50 μM), then incubated for a duration of 4 days at 30 °C. Subsequently, the colony surface was treated with sterile water and 2.5% bromophenol blue solution for 12 h and then photographed.

### 5.5. Effect of Rhein on A. flavus Cell Wall

*A. flavus* spore suspension was inoculated into PDA medium supplemented with rhein (50 μM) and fluorescent brightener 28 (CFW, 100 and 200 μg/mL) and incubated at 30 °C for 3 days. Moreover, the *A. flavus* colony diameter was recorded and photographed to evaluate the effect of rhein on *A. flavus* cell walls.

*A. flavus* spore suspension and rhein (25 and 50 μM) were added to 50 mL DPY medium and incubated for 48 h. Following the staining method described in a previous study [58], the mycelia were collected, washed, and then stained with Calcofluor white stain (CFW) for 1 min in the dark on slides. Finally, confocal laser scanning microscopy (CLSM) was utilized to observe the mycelia.

### 5.6. Effect of Rhein on A. flavus Cell Membrane

*A. flavus* spore was point-inoculated onto PDA supplemented with 50 μM rhein and different concentrations of sodium dodecyl sulphate (SDS, 100, and 200 μg/mL), and then it was incubated at 30 °C for 4 days. Then, the colony diameters were assessed and the relative inhibition rate was calculated to evaluate the effect of rhein on inhibition of *A. flavus* cell membranes.

Similarly, *A. flavus* mycelia were treated as described above. *A. flavus* spore suspension and rhein (25 and 50 μM) were added to 50 mL DPY medium and allowed to grow for 48 h. The mycelia were then collected and washed. Subsequently, propidium iodide (PI) staining was carried out at 37 °C for 30 min. Finally, the mycelia were collected and examined through CLSM.

### 5.7. Determination of A. flavus Intracellular ROS, H_2_O_2,_ and ATP Content

*A. flavus* spore suspension and various rhein concentrations (25 and 50 μM) were added to 50 mL DPY medium and allowed to grow for 48 h at 30 °C. The mycelia were treated as described above, then stained with DCFH-DA and incubated in the dark at 37 °C for 30 min. Finally, samples were visualized using CLSM.

*A. flavus* spore was point-inoculated on PDA medium with cellophane with or without rhein (50 μM) and incubated at 30 °C for 60 h. Subsequently, *A. flavus* mycelia were ground into powder and collected. The H_2_O_2_ and ATP contents were determined with reference to the biochemical kit (Solarbio Science, Beijing, China).

### 5.8. Effect of Rhein on A. flavus Mycelium Growth and AFB_1_ Yield on Peanuts and Maize

Different concentrations of rhein (25 and 50 μM) and *A. flavus* spore suspension were added into 50 mL sterile water until a final concentration of 1 × 10^6^ spores/mL was achieved. Then, sterilized peanuts and corn were placed in the water and shaken for 1 h. The treated peanuts and corn were then evenly placed in a dish covered with filter paper and cultured at 30 °C in the dark for 5 days. To maintain appropriate moisture levels, sterile water was added periodically during this incubation period. The infected peanut and corn seeds were placed in triangular flasks after 5 days of incubation, and the spores were counted after 30 min of thorough agitation after adding 20 mL of sterile water. AFB_1_ was extracted and determined as described above.

### 5.9. Statistical Analysis

In this study, all experiments were performed in triplicate and presented as mean ± SD, followed by significance analysis using SAS software (version 9.2), with statistical significance considered at *p* < 0.05.

## Figures and Tables

**Table 1:** The representative DEGs in the control compared to the rhein treatment.

Gene ID	log_2_(FC)	Gene Functional Description
Conidium formation and development		
*AFLA_052030*	−1.55	Developmental regulatory protein wetA
*AFLA_082850*	−2.47	C2H2 type conidiation transcription factor BrlA
*AFLA_029620*	−2.06	Transcription factor AbaA
*AFLA_098380*	−1.85	Conidial hydrophobin RodA
*AFLA_014260*	−1.44	Conidial hydrophobin RodB
*AFLA_044790*	−1.63	Conidiation-specific family protein
Cell wall and membrane		
*AFLA_052780*	−1.60	Cell wall glucanase (Scw4)
*AFLA_061960*	−1.94	Cell wall protein
*AFLA_137200*	1.56	Chitin synthase
*AFLA_025900*	−2.71	Chitin deacetylase
*AFLA_007630*	−1.52	Integral membrane protein
*AFLA_039810*	−1.23	DUF1212 domain membrane protein
Oxidative stress		
*AFLA_096210*	−1.79	Catalase
*AFLA_034380*	−1.28	Catalase
*AFLA_071780*	−2.42	Peroxidase family protein
*AFLA_001980*	−1.45	Glutamate cysteine ligase
Glycolysis, citrate cycle, and aflatoxin biosynthesis		
*AFLA_101470*	1.05	Glyceraldehyde-3-phosphate dehydrogenase
*AFLA_036370*	−1.29	Phosphoenolpyruvate carboxykinase
*AFLA_107660*	−2.13	Succinyl-CoA synthetase
*AFLA_117410*	−4.26	Citrate synthase
*AFLA_117420*	−1.75	Fatty acid synthase subunit alpha (aflA)
*AFLA_081850*	−1.23	Norsolorinic acid synthase (aflC)
*AFLA_065570*	−1.35	O-methyltransferase (aflO)
*AFLA_073130*	1.28	O-methylsterigmatocystin oxidoreductase ordA-like (aflQ)

## Data Availability

The raw transcriptome read data are available in the CNCB database under accession number CRA015824.
